# The Free Hormone Hypothesis: When, Why, and How to Measure the Free Hormone Levels to Assess Vitamin D, Thyroid, Sex Hormone, and Cortisol Status

**DOI:** 10.1002/jbm4.10418

**Published:** 2020-11-02

**Authors:** Daniel D Bikle

**Affiliations:** ^1^ Department of Medicine University of California San Francisco USA; ^2^ Department of Medicine San Francisco VA Medical Center San Francisco CA USA

**Keywords:** BINDING PROTEINS, CORTISOL, FREE HORMONE HYPOTHESIS, TESTOSTERONE, THYROID HORMONE, VITAMIN D

## Abstract

The free hormone hypothesis postulates that only the nonbound fraction (the free fraction) of hormones that otherwise circulate in blood bound to their carrier proteins is able to enter cells and exert biologic effects. In this review, I will examine four hormone groups—vitamin D metabolites (especially 25OHD), thyroid hormones (especially thyroxine [T4]), sex steroids (especially testosterone), and glucocorticoids (especially cortisol)—that are bound to various degrees to their respective binding proteins—vitamin D‐binding protein (DBP), thyroid‐binding globulin (TBG), sex hormone‐binding globulin (SHBG), and cortisol‐binding globulin (CBG)—for which a strong case can be made that measurement of the free hormone level provides a better assessment of hormonal status than the measurement of total hormonal levels under conditions in which the binding proteins are affected in levels or affinities for the hormones to which they bind. I will discuss the rationale for this argument based on the free hormone hypothesis, discuss potential exceptions to the free hormone hypothesis, and review functions of the binding proteins that may be independent of their transport role. I will then review the complications involved with measuring the free hormone levels and the efforts to calculate those levels based on estimates of binding constants and levels of both total hormone and total binding protein. In this review, the major focus will be on DBP and free 25OHD, but the parallels and differences with the other binding proteins and hormones will be highlighted. Vitamin D and its metabolites, thyroid hormones, sex steroids, and glucocorticoids are transported in blood bound to serum proteins. The tightness of binding varies depending on the hormone and the binding protein such that the percent free varies from 0.03% for T4 and 25OHD to 4% for cortisol with testosterone at 2%. Although the major function of the primary carrier proteins (DBP, TBG, SHBG, and CBG) may be to transport their respective lipophilic hormones within the aqueous media that is plasma, these proteins may have other functions independent of their transport function. For most tissues, these hormones enter the cell as the free hormone presumably by diffusion (the free hormone hypothesis), although a few tissues such as the kidney and reproductive tissues express megalin/cubilin enabling by endocytosis protein‐bound hormone to enter the cell. Measuring the free levels of these protein‐bound hormones is likely to provide a better measure of the true hormone status than measuring the total levels in situations where the levels and/or affinities of the binding proteins are altered. Methods to measure free hormone levels are problematic as the free levels can be quite low, the methods require separation of bound and free that could disturb the steady state, and the means of separating bound and free are prone to error. Calculation of free levels using existing data for association constants between the hormone and its binding protein are likewise prone to error because of assumptions of linear binding models and invariant association constants, both of which are invalid. © 2020 The Author. *JBMR Plus* published by Wiley Periodicals LLC on behalf of American Society for Bone and Mineral Research.

## Introduction

Lipophilic hormones such as steroid hormones including vitamin D and thyroid hormone are transported in the aqueous media such as plasma by specific and nonspecific proteins. The specific proteins include vitamin D‐binding protein (DBP), which binds vitamin D and its metabolites; thyroid‐binding globulin (TBG), which binds thyroxine (T4) and triiodothyronine (T3); sex hormone‐binding globulin (SHBG), which binds testosterone and estradiol and other sex steroids; and cortisol‐binding globulin (CBG), which binds cortisol, aldosterone, and progesterone. Nonspecific‐binding proteins include albumin and lipoproteins. In the case of T4, transthyretin also contributes significantly to the binding. The fraction of hormone that is not bound to its respective carrier proteins is the free fraction. The free fraction is determined primarily by both the level and affinity of the specific binding protein, although the large amount of albumin in blood contributes to this determination. Table 1 lists the association constants that have been reported for the hormones and binding proteins discussed in this review, although these values are influenced both by clinical circumstances and mutations in the binding proteins. Of particular interest is that the free fraction varies from approximately 0.03% for T4 and 25OHD, 2% for testosterone, and 4% to 10% for cortisol. Interest in knowing the free hormone levels is based on the concept that in the case of these hormones only the free fraction is able to cross the plasma membrane into the target cells. This is the free hormone hypothesis. A corollary of this hypothesis is that measurement of the free hormone level is necessary to determine the true hormonal state. A powerful argument in support of this concept is provided by families in which the gene for the binding protein of interest is mutated rendering it null, yet the affected family members show little clinical evidence of hormone deficiency. Nevertheless, this concept has proven controversial, but at least for T4, measurement of free T4 along with thyroid‐stimulating hormone (TSH) has replaced measurement of total T4 in assessment of thyroid status. Similar arguments supporting the routine use of free hormone measurements will be presented in this review.

**Table 1:** Binding Proteins for the Vitamin D Metabolites, Thyroid Hormones, Sex Steroids, and Glucocorticoids/Mineralcorticoids

Hormone	Specific BP	Ka	% Bound	Albumin	Ka HAS	% bound	TTR	Ka TTR	% Bound	% Free	Reference
25OHD	DBP	7–9 × 10^8^	88	HSA	6 × 10^5^	12				0.02–0.04	Bikle et al, 1986^(^ ^42^ ^)^
1,25(OH)_2_D	DBP	4 × 10^7^	85	HSA	5.4 × 10^4^	15				0.3–0.4	Bikle et al, 1985^(^ ^44^ ^)^
T4	TBG	1 × 10^10^	70	HSA	1.5 × 10^6^	5	TTR	2 × 10^8^	20	0.03	Mimoto & Refetoff, 2020^(^ ^60^ ^)^
T3	TBG	1 × 10^9^	75	HSA	2 × 10^5^	20	TTR	1 × 10^6^	5	0.3	Mimoto & Refetoff, 2020^(^ ^60^ ^)^
Testosterone	SHBG	2 × 10^9^	44	HSA	4 × 10^4^	50				2	Heyns, 1971
Estradiol	SHBG	7 × 10^8^	20	HSA	6 × 10^4^	78				2	Heyns, 1971^(^ ^73^ ^)^
Cortisol	CBG	8 × 10^7^	90	HSA	3 × 10^3^	7				4	Stroupe et al, 1978^(^ ^92^ ^)^
Aldosterone	CBG	2 × 10^6^	37	HSA	2 × 10^3^	21				37	Dunn et al, 1981^(^ ^93^ ^)^

This table shows the binding constants of these binding proteins for their respective hormones under normal conditions, the % of the total hormone level bound to the protein, and the % free. Numerous clinical conditions and genetic modifications alter these numbers, which are shown here for comparative purposes. BP = binding protein; CBG = cortisol‐binding globulin; DBP = vitamin D‐binding protein; HAS = human serum albumin; Ka = association constant; SHBG = sex hormone‐binding globulin; T3 = triiodothyronine; T4 = thyroxine; TBG = thyroid‐binding globulin; TTR = transthyretin.

## The Free Hormone Hypothesis

### The hypothesis and its modification

**Fig 1:**
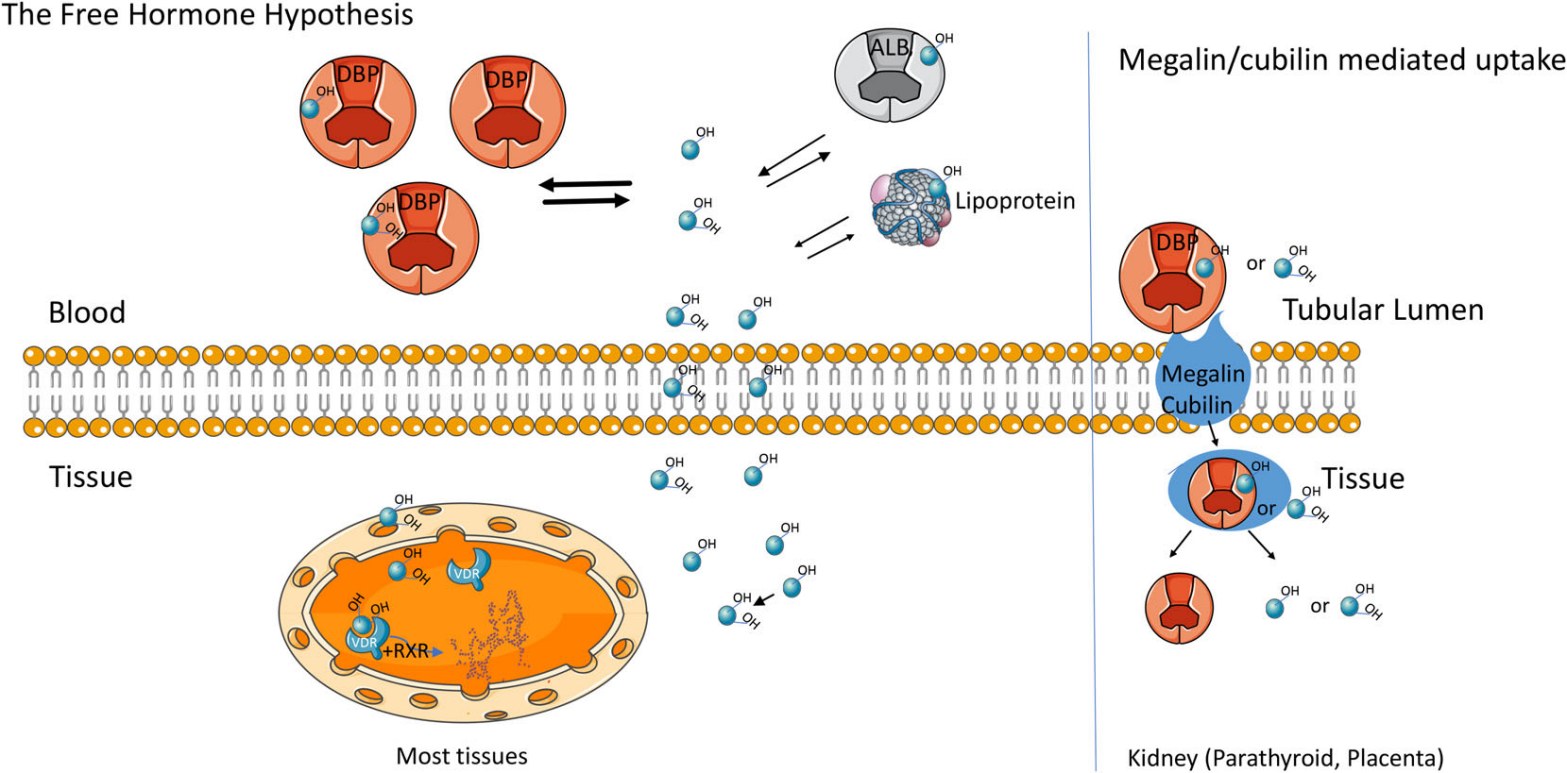
The free hormone hypothesis as illustrated for vitamin D metabolites. Vitamin D metabolites (blue circles) in the circulation are tightly bound to vitamin D‐binding protein (DBP) and to a lesser extent to albumin (ALB). For most tissues, only the unbound metabolites can cross the cell membrane. In several tissues such as the kidney and potentially the parathyroid and placenta 25OHD and 1,25(OH)_2_D bound to DBP may enter cells by endocytosis via megalin/cubilin and are not limited to diffusion by the free hormone. Figure reproduced from Bikle DD, Malmstroem S, Schwartz J. Current controversies: are free vitamin metabolite levels a more accurate assessment of vitamin D status than total levels? Endo Clin NA. 2017;46:901–18.

The free hormone hypothesis postulates that only the nonbound fraction (the free fraction) of total hormones that otherwise circulate in blood bound to their carrier proteins is able to enter cells and exert its biologic effects (Figure 1). Examples include the vitamin D metabolites, thyroid hormone, sex steroids, and cortisol. These are lipophilic hormones assumed to cross the plasma membrane by diffusion and not by an active transport mechanism. Perhaps the earliest articulation of the free hormone hypothesis was published by Recant and Riggs^(^ ^1^ ^)^; they examined thyroid function in patients with protein‐losing nephropathy. They noted that circulating thyroid hormone (measured as protein‐bound iodine) was quite low in these patients along with increased urinary losses, but with relatively little evidence for clinical hypothyroidism. They concluded that “thyroid function and the supply of hormone to the tissues in nephrosis may be normal, and that the low concentration of protein‐bound iodine in the plasma is due to the change in concentration or binding capacity of the plasma proteins in nephrosis.” Indeed, one of the strongest arguments favoring the free hormone hypothesis is that subjects with mutations in the genes for their respective binding proteins that alter the affinity or levels of the binding protein for the hormone such that total hormone levels are quite reduced have limited clinical impact, despite the low total hormone levels that might otherwise be expected in hormone deficiency. Examples will be detailed subsequently when individual binding proteins are discussed.

However, the free hormone concentration is not the only factor involved with the rate at which the hormone enters the cells. As articulated by Mendel,^(^ ^2^ ^)^ the movement of hormone into the cell in vivo is dependent not only on the free concentration, but also on the dissociation of hormone from its binding proteins, the rate of blood flow, the rate of uptake into the cell, and the catabolism/sequestration of hormone within the cell. These are components of the transport process that Mendel calls the free hormone transport process. Thus, the total concentration of hormone, by affecting the total amount of free hormone available to the cell, does influence the extent of transport of hormone into the cell when the rate of transport is not rate‐limiting. These considerations also reflect upon what is called the bioavailable hormone, generally that percent of hormone bound to albumin as well as free. Although the affinity constants of albumin for the hormones we are discussing are lower than that for the specific binding proteins, it is not clear that the dissociation of the hormone from albumin is not rate‐limiting for any and all target tissues, so suggesting that the so‐called bioavailable fraction is truly bioavailable is not established for any of the hormones we will discuss. Indeed at least in vitro albumin does restrict entry of these hormones into cells where examined.^(^ ^3^ ^)^ Our knowledge of most of the variables involved with transport into cells in vivo is limited, so our focus in this review will be on the free hormone itself, its measurement, and what influences this fraction of the total hormone in circulation.

### The megalin/cubilin transport system

As noted above, the free hormone hypothesis postulates that only the free hormone can cross the plasma membrane. An important exception exists in some tissues for some of the protein‐bound hormones. The renal tubule differs from most other tissues in its mechanism for at least 25OHD uptake, and likely for all DBP‐bound vitamin D metabolites. The DBP–25OHD complex is filtered in the glomerulus and reabsorbed in the proximal tubule through endocytosis by the megalin/cubilin complex (Fig. 1), thereby providing 25OHD for CYP27B1 1α‐hydroxylation in the kidney tubule, as well as for the rest of the body.^(^ ^4^, ^5^ ^)^ This complex is not specific for DBP, but when megalin is deleted, the major protein lost in the urine is DBP. In the mice that survive long enough, bone growth is retarded and osteopenic.^(^ ^4^ ^)^ The impact of cubilin deletion is similar, but not as severe.^(^ ^5^ ^)^ A similar mechanism may operate in the parathyroid gland and placenta, which like the renal tubule express megalin/cubilin,^(^ ^6^ ^)^ but at this point experiments to determine the impact of either megalin or cubilin deletion from the parathyroid or gland or placenta have not been reported. Similarly, megalin has been found in reproductive tissues where it enables endocytosis of sex steroids bound to SHBG. Male mice lacking megalin have undescended testes, whereas female mice have failure of vaginal opening.^(^ ^7^ ^)^ On the other hand, although there may be some uptake of DBP by immune cells, this does not appear to involve megalin/cubilin.^(^ ^8^ ^)^ The role of megalin/cubilin in the uptake of other protein bound hormones has not been described to my knowledge.

## Vitamin D‐Binding Protein and Free Vitamin D Metabolites

As shown in Table 1, DBP has one of the highest association constants for 25OHD, comparable to that of TBG for T4, resulting in one of the lowest free fractions. In serum samples from normal individuals, approximately 88% of circulating 25OHD and 85% of 1,25(OH)_2_D are bound to DBP, whereas albumin with its substantially lower binding affinity binds only approximately 12% to 15% of these metabolites despite its 10‐fold higher concentration than DBP. Approximately 0.4% of total 1,25(OH)_2_D and 0.03% of total 25OHD is free in serum from normal nonpregnant individuals. The degree of saturation of DBP by the vitamin D metabolites is very low (DBP circulates at micromolar levels, whereas 25OHD concentrations are nanomolar) such that changes in vitamin D levels have little impact on the free fraction. This is not true for CBG or SHBG as will be discussed. However, vitamin D intoxication has been shown to have a modest increase in the free fraction.^(^ ^9^ ^)^ Nevertheless, changes in the level of DBP clearly do affect the free fraction as will be discussed.

### Genomic regulation

The human DBP gene is located on chromosome 4q12‐q13 in close association with other members of the albuminoid family: albumin, α‐fetal protein, and afamin. It is 35 kb in length and comprised of 13 exons encoding 474 amino acids, including a 16 amino acid leader sequence, which is cleaved before release. Numerous tissues express DBP, but the liver is the major source.^(^ ^10^ ^)^ The expression of DBP is increased by estrogen, but not by androgens,^(11^ ^)^ as appreciated with the rise in DBP during pregnancy^(^ ^12^, ^13^ ^)^ and with oral contraceptive administration.^(^ ^14^ ^)^ Dexamethasone and certain cytokines such as IL‐6 also increase DBP production, whereas TGFβ is inhibitory.^(^ ^15^ ^)^ These cytokines and glucocorticoids are likely to play a role in the increase in DBP production following trauma^(^ ^16^ ^)^ and acute liver failure.^(^ ^17^ ^)^ Primary hyperparathyroidism, on the other hand, is associated with a reduction in DBP levels, likely contributing to the lower 25OHD levels in these patients as the free 25OHD is not reduced.^(^ ^18^ ^)^ Vitamin D itself or any of its metabolites does not regulate DBP production.^(^ ^19^ ^)^

### Structure and polymorphisms

The mature human DBP is approximately 58 kD in size, although differences in glycosylation of the protein for different alleles alter the actual size. DBP is the most polymorphic gene known. Over 120 variants have been described based on electrophoretic properties^(^ ^20^ ^)^ with 1242 polymorphisms currently listed in the National Center for Biotechnology Information database.^(^ ^21^ ^)^ Of these variants, the Gc1f and Gc1s (rs7041 locus) and Gc2 (rs4588 locus) are the most common. The Gc1f and Gc1s involve two polymorphisms, one at aa 432 (416 in the mature DBP) and 436 (420 in the mature DBP). The 1f allele encodes the sequence of aa between 432–436 as DATPT, the 1s allele encodes the sequence EATPT. This subtle difference in charge makes Gc1f run faster (fast) than the Gc1s (slow) during electrophoresis. The Gc2 allele encodes DATPK, which runs slower still. Glycosylation further distinguishes the Gc1 variants from the Gc2 variant. The threonine (T) in Gc1 binds N‐acetylgalactosamine to which galactose and sialic acid bind in tandem. The lysine (K) in a comparable position in Gc2 is not glycosylated.^(^ ^22^, ^23^ ^)^ This affects the conversion of DBP to DBP–MAF (macrophage activating factor), which involves a partial deglycosylation removing the galactose and sialic acid by the sequential action of sialidase and β‐galactosidase by T and B cells.^(^ ^24^ ^)^ The SNPs are in complete linkage disequilibrium, and only six haplotypes are observed with any significant frequency. Gc2 is the least abundant and Gc1f the most abundant. Gc alleles show distinct racial distribution patterns. Black and Asian populations are more likely to carry the Gc1f form and the Gc2 form is rare, whereas White populations more frequently exhibit the Gc1s and the Gc2 form. Gc1f has been stated to have the highest affinity and Gc2 the lowest affinity for vitamin D and its metabolites,^(^ ^25^ ^)^ but these differences among alleles have not been found by others,^(26^ ^)^ and account for little of the variation seen among various populations that we recently reported.^(^ ^27^ ^)^

DBP is comprised of three structurally similar domains. The first domain is the binding site for the vitamin D metabolites (aa 35‐49). Fatty acid binding utilizes a single high‐affinity site for both palmitic acid and arachidonic acid, but only arachidonic acid competes with 25OHD for binding.^(^ ^28^, ^29^ ^)^ The actin‐binding site is located at aa 373‐403, spanning parts of domains 2 and 3, but part of domain 1 is also involved.^(^ ^30^, ^31^ ^)^ The C5a/C5a des Arg binding site is located at aa 130‐149.^(^ ^32^ ^)^ Membrane binding sites have been identified in aa 150‐172 and 379‐402.^(^ ^33^ ^)^ Less clear is the role of these sites in facilitating transport of the vitamin D metabolites into the cell, and if so whether polymorphisms in these or other sites affect that transport. In the absence of disease or pregnancy, DBP levels are relatively constant over time in adults.^(^ ^34^ ^)^ That said, various substances in the blood such as polyunsaturated fatty acids may alter the affinity of DBP for the vitamin D metabolites.^(^ ^29^ ^)^

### Biologic function

The major role of DBP with respect to vitamin D is its transport function. DBP was discovered by Hirschfeld in 1959,^(^ ^35^ ^)^ and originally called group‐specific component (Gc‐globulin), but it was not until 1975 that its function as a vitamin D transport protein was appreciated.^(^ ^36^ ^)^ As noted above in normal individuals, approximately 85% of circulating vitamin D metabolites are bound to DBP, albumin binds approximately 15% of these metabolites such that approximately 0.4% of total 1,25(OH)_2_D_3_ and 0.03% of total 25(OH)D_3_ is free in serum from normal nonpregnant individuals. The affinity of DBP for the vitamin D_2_ metabolites is somewhat less than that for the vitamin D_3_ metabolites.^(^ ^37^ ^)^ The importance of this transport mechanism with respect to access of the vitamin D metabolite to the cell is well‐illustrated in the DBP KO mouse. In these mice, the vitamin D metabolites are presumably all free and/or bioavailable as albumin levels are normal. Tissue levels of 1,25(OH)_2_D were normal in the DBP KO mice, and markers of vitamin D function such as expression of intestinal TRPV6, calbindin 9k, PMCA1b, and renal TRPV5 were maintained despite having very low levels of serum 25OHD and 1,25(OH)_2_D and increased loss of these metabolites in the urine.^(^ ^38^ ^)^ Moreover, injection of 1,25(OH)_2_D into these DBP KOs showed a more rapid increase in the expression of Cyp24A1, TRPV5, and TRPV6 than in DBP intact controls.^(^ ^39^ ^)^ However, on a vitamin D‐deficient diet, they quickly developed vitamin D deficiency. Recently, a family has been reported in which the proband had a homozygous mutation in DBP with undetectable serum levels of DBP and 25OHD and very low levels of 1,25(OH)_2_D.^(^ ^40^ ^)^ PTH and serum calcium were normal. The proband had osteopenia and ankylosing spondylitis. Her seven siblings did not have evidence of bone disease or altered calcium metabolism, including her heterozygote brother with low levels of DBP and total levels of 25OHD, but normal levels of free 25OHD. Thus, DBP appears not to be required for access of the vitamin D metabolites to cells, consistent with the free hormone hypothesis, but serves as a circulating reservoir for the vitamin D metabolites reducing the risk of vitamin D deficiency when intake or epidermal production is limited. DBP has other important functions including actin scavenging, neutrophil recruitment, and macrophage recruitment reviewed previously,^(41^ ^)^ but a discussion of which is outside the scope of this review.

### Directly measured free vitamin D metabolites

The very low levels of free 25OHD and 1,25(OH)_2_D make their measurement a challenge. Equilibrium dialysis followed by measurement of the free hormone in the dialysate via liquid chromatography/mass spectrometry is generally considered the gold standard, but technical difficulties, labor, and cost make this an unsuitable technique to use for the vitamin D metabolites. We adopted centrifugal ultrafiltration dialysis to obtain the data enabling us to determine binding constants for DBP and albumin and the free fraction for both 25OHD and 1,25(OH)_2_D shown in Table 1.^(^ ^42^, ^43^, ^44^, ^45^ ^)^ Moreover, these studies enabled us to demonstrate the changes in the free fraction associated with changes in DBP levels during liver disease and pregnancy, data indicating the important contribution that measurement of the free hormone level could make in assessing hormone status in conditions in which DBP levels varied. However, this method is not suited to high throughput, and free vitamin D measurements were not readily available until an immunometric method came onto the market. At this point, no alternative method to measure free 1,25(OH)_2_D has been developed.

#### ELISA method

A two‐step ELISA that directly measures free 25OHD levels has been developed (Future Diagnostics Solutions B.V., Wijchen, The Netherlands). In the first incubation step, an antivitamin D monoclonal antibody immobilized on a microtiter plate binds the free 25OHD in the serum sample. After washing away excess serum, the second incubation step is to add biotinylated 25OHD in a known amount to react with the unoccupied binding sites on the monoclonal antibody attached to the plate. The nonbound biotinylated 25OHD is then removed by a second washing. Thereafter, streptavidin peroxidase conjugate is added followed by the substrate 3,3′,5,5′‐tetramethylbenzidine. The bound streptavidin peroxidase can be quantified by measuring the absorbance at 450 nm generated in the reaction spectrophotometrically. The intensity is inversely proportional to the level of free 25OHD. The limit of detection is 2.8 pg/mL. This assay is dependent on the quality of the antibody used to bind the free 25OHD. The antibody in the current assay does not recognize 25(OH)D_2_ as well as 25(OH)D_3_ [77% of the 25(OH)D_3_ value], so it underestimates the free 25(OH)D_2._ However, under most situations where the predominant vitamin D metabolite is 25(OH)D_3_, the data compare quite well to those obtained from similar populations using the centrifugal ultrafiltration assay.^(^ ^26^, ^46^ ^)^

#### Liquid chromatography–tandem mass spectrometry method

Liquid chromatography–tandem mass spectrometry (LC–MS/MS) has been used to detect 25OHD in saliva, which is expected to be free of DBP and albumin and so represents free 25OHD.^(^ ^47^ ^)^ In this method, 1 mL of saliva is deproteinized with acetonitrile, purified using a Strata‐X cartridge, derivatized with 4‐phenyl‐1,2,4‐triazoline‐3,5 dione, ionized by electron spray ionization, and subjected to LC–MS/MS. This method has not received widespread use.

### Calculation of free vitamin D metabolites

Because of the difficulty in measuring the free hormone, some investigators have used the association constants of DBP and albumin for the vitamin D metabolites along with measurements of total metabolite, DBP, and albumin levels to calculate the free fraction according to the following formula:

$$\text{Free vitamin}\;D\;\text{metabolite}=\frac{\text{Total vitamin}\;D\;\text{metabolite}}{1+\left({\mathrm{Ka}}_{\mathrm{alb}}*\text{albumin}\right)+\left({\mathrm{Ka}}_{\mathrm{DBP}}*\mathrm{DBP}\right)}$$

*Ka* is the association constant. This calculation makes a number of assumptions, including constancy of the affinity constants from individual to individual in all clinical conditions; linearity of binding to the binding proteins at all hormone concentrations; and accurate measurement of DBP, albumin, and the vitamin D metabolite of interest. All of these assumptions have proved problematic, although initial studies in normal subjects, in which DBP levels were measured with a polyclonal assay, the calculated values agreed reasonably well with the directly measured values using the centrifugal ultrafiltration dialysis method.^(^ ^42^, ^45^ ^)^ However, with different populations, different assays of DBP and 25OHD, and different clinical situations the calculated levels have not correlated well with the directly measured levels, and use of this formula to calculate the free hormone level cannot be recommended.^(^ ^48^ ^)^

### Free 25OHD measurements in different populations and clinical conditions

#### PTH and vitamin D

Directly measured free 25OHD concentrations are strongly correlated with total 25OHD concentrations and have been reported to be between 0.02% and 0.09% of total 25OHD concentrations.^(^ ^27^ ^)^ Concentrations generally range from 1.2 to 7.9 pg/mL. PTH is negatively correlated with free 25OHD, as well as total 25OHD. Serum C‐terminal telopeptide of type I collagen has been reported to have a moderate positive correlation with total and free 25OHD.^(^ ^49^ ^)^ With vitamin D supplementation, free 25OHD concentrations rise in concert with total 25OHD concentrations,^49^, ^50^, ^51^, ^52^ ^)^ rising more steeply with vitamin D_3_ supplementation compared with vitamin D_2._^(^ ^53^ ^)^ With high0dose vitamin D supplementation, the changes in iPTH were significantly related to changes in free 25OHD, but not to changes in total 25OHD or changes in total 1,25(OH)_2_D.^(^ ^53^ ^)^

#### Race

Although DBP levels in a Black population were thought to be lower using a monoclonal antibody, resulting in a normal calculated free 25OHD despite the low total 25OHD, these results were subsequently shown to result from the use of a monoclonal antibody that failed to efficiently measure the dominant G1f allele in the Black population.^(^ ^54^ ^)^ Subsequent studies with a polyclonal assay found similar DBP levels between Black and White populations and consistently lower free 25OHD levels in the Black population, consistent with lower total 25OHD.^(^ ^55^, ^56^ ^)^ In general, differences in DBP genotype has had little impact on free 25OHD levels, although the G2 genotype is consistently associated with slightly lower total and free 25OHD.^(^ ^27^ ^)^

#### Sex and pregnancy

Investigations have not found differences in free levels of 25OHD between normal men and women relative to total 25OHD levels.^(27^ ^,51^ ^)^ Estrogen administration raises both the total and free 1,25(OH)_2_D levels, along with DBP levels, without significant changes in the free fraction.^(^ ^57^, ^58^ ^)^ The data regarding the impact of estrogen administration on free 25OHD are less clear as they are cross sectional derived from subjects on oral contraceptives, which generally contain a progestin that, at least in one study, blocks the rise in free 1,25(OH)_2_D, perhaps also blocking the increase in free 25OHD.^(^ ^58^ ^)^ However, directly measured free 25OHD tends to be higher, and free 1,25(OH)_2_D is substantially higher in pregnant women versus comparator groups of women.^(^ ^45^, ^48^ ^)^ Because these results, at least for free 1,25(OH)_2_D, are nonlinear with respect to the parallel increase in DBP, they suggest that the affinity of DBP for 1,25(OH)_2_D is decreased during pregnancy, and could account for the increased intestinal calcium transport observed during the last trimester, when demands for calcium by the developing fetus are greatest.

#### Liver disease

Directly measured free 25OHD and 1,25(OH)_2_D are higher in outpatients with cirrhosis compared with other groups,^43^, ^48^ ^)^ despite lower total vitamin D metabolite concentrations. The relationship between free 25OHD and total 25OHD is both steeper and more variable in patients with liver disease than in healthy people.^(^ ^27^ ^)^ Those with the most‐severe cirrhosis and protein synthesis dysfunction have a higher percentage of free 25OHD compared with cirrhotics without protein synthesis dysfunction, but free 25OHD concentrations are similar because of the presence of both lower total 25OHD concentrations as well as lower DBP.^(^ ^59^ ^)^ Free 25OHD concentrations range between 4.5 to 8.1 pg/mL in cirrhotics with low albumin and from 6.4 to 10.6 pg/mL in those with normal albumin.

#### Frail elderly

Nursing home residents are older, have more medical problems, receive more medications, and are more likely to have poorer nutrition than younger people or community‐dwelling elderly. In vitamin D dose‐titration studies,^(^ ^52^ ^)^ free 25OHD levels rose in response to increases in total 25OHD, but responses appeared to be steeper than those of normal subjects despite comparable DBP levels,^(^ ^27^ ^)^ suggesting that in this group of subjects the affinity of DBP for 25OHD is reduced perhaps because of interfering medications or low‐grade inflammatory states, although this remains speculative.

## Thyroid Hormone‐Binding Proteins and Free Thyroid Hormones

The status of thyroid sufficiency is generally assessed by a combination of TSH and the free levels of T4 because alterations in the thyroid hormone‐binding proteins (THBPs) are relatively common, and the free hormone levels are more accurate indicators of thyroid status than the total hormone (TH) levels. For the thyroid hormones there are three major THBPs: thyroid‐binding globulin (TBG), which has the highest binding affinity for the TH; transthyretin (TTR; formerly known as prealbumin and renamed in recognition of its role in retinol binding: TRANSport of THYroid hormone and RETINol), and albumin.^(^ ^60^ ^)^ The affinity constants, approximate amounts of T4 and T3 bound, and percent of free levels are given in Table 1. As noted previously, TBG and DBP have comparable high affinity for their respective hormones, such that the free hormone concentrations are quite low. In the case of TBG, approximately 75% of T4 and T3 are bound to the protein. Although free T4 and free T3 are recognized as the form entering the cells (free hormone hypothesis), the minor component bound to lipoproteins may have facilitated entry to cells expressing the LDL receptor.^(^ ^61^ ^)^ Unlike the vitamin D metabolites, there are known transporters for the TH in the cell membranes: monocarboxylate transporters MCT8 and 10, L‐amino acid transporters LAT1 and 2, and the organic anion transporter peptide OATP1C1.^(^ ^62^ ^)^ Although mutations in the THBPs, including null mutations in TBG, do not affect the euthyroid status of the subject despite marked changes in total TH levels consistent with the free hormone hypothesis, such mutations, at least for TTR, can cause other morbidities.

### Thyroid‐binding globulin

TBG is a 54 kDa 395 amino acid monomer with a single binding site for TH. It has four glycosylation sites. Estrogen increases TBG levels primarily by increasing the sialic acid content of these carbohydrate chains increasing its half‐life in the blood. Androgens have the opposite effect.^(^ ^63^ ^)^ TBG levels are also reduced by glucocorticoids,^(^ ^64^ ^)^ hepatic failure, severe nonthyroidal illness, protein‐losing nephropathies, or gastrointestinal disorders.^(^ ^65^ ^)^ Its gene is located on the X chromosome, such that mutations typically have greater effects on males than females. The promoter has binding sites for hepatic nuclear factors 1 and 3, and the liver is the major site of production. There are 28 TBG mutations that result in complete loss of function generally from gene deletions, 18 partial deficiencies resulting primarily from missense mutations, and several gene duplications that increase TBG levels (reviewed in Mimoto and Refetoff^(^ ^60^ ^)^). Although these mutations have profound effects on total TH levels, the free TH levels are normal and the subjects are euthyroid. That said, these THBGs do appear to alter tissue distribution of TH as shown in liver perfusion studies by Mendel.^(^ ^66^ ^)^

### Transthyretin

Unlike TBG, TTR is expressed in multiple tissues. Its gene is located on chromosome 18, encoding a 55 kDa 127 amino acid protein that circulates as a homo tetramer. This complex has two binding sites for TH, but the second site in under negative cooperativity such that the actual affinity for TH depends to some degree on TH concentrations. As mentioned, TTR is expressed in multiple tissues including the choroid plexus. As such, TTR is the dominant THBG in cerebrospinal fluid.^(^ ^67^ ^)^ Like TBG, a number of mutations of TTR have been reported, none of which alter free TH levels or thyroid status. However, many of these mutations lead to amyloidosis (reviewed in Mimoto and Refetoff ^(60^ ^)^).

### Albumin

Human serum albumin (HSA) is a 66.5 kDa 585 amino acid protein made in the liver. Although HSA binds all the hormones being discussed in this review, as noted in Table 1, it is described in this section because of a common set of mutations that increase its affinity for T4, T3, or both. This results in the syndrome of familial dysalbuminemic hyperthyroxinemia (FDH).^(^ ^68^ ^)^ This leads to elevated levels of total T4 and/or T3, but normal levels in free TH. It is quite common in Hispanics (up to 1:50 of those with Puerto Rican ancestry).^(^ ^69^ ^)^ Other albumin mutations, including analbuminemia (total lack of albumin), likewise have little impact on thyroid status.^(^ ^70^ ^)^

#### Measurement of free total hormone

As for the measurement of the free vitamin D metabolites, the very low levels of the free TH makes this challenging. Equilibrium dialysis followed by LC–MS/MS is the gold standard, but is not practical for high throughput. Accordingly, measurements of free T4 and free T3 rely on competitive immunoassays in which the free hormone is first extracted from blood with specific antibodies, incubated with a labeled TH probe, and the unoccupied antibody binding sites determined, which are inversely proportional to the free hormone,^(71^ ^)^ similar to the method described for free 25OHD. Two‐step methods are preferable because they do not require the use of T4 analogs and are less prone to nonspecific binding.^(^ ^72^ ^)^ These methods are susceptible to a number of problems with heterophile antibodies, biotin consumption by the subject, and antibody specificity. Patients with mutations in albumin that increase the binding to TH, such as FDH, can show spuriously elevated free TH levels with the automated immunoassays.^(^ ^60^ ^)^

## Sex Hormone‐Binding Globulin and Free Testosterone

Sex hormone‐binding globulin (SHBG) is the most specific, highest affinity protein in blood for testosterone, dihydrotestosterone, and estrogen. It binds approximately 44% of total testosterone and 20% estradiol, whereas the lower affinity, but much more abundant, albumin binds 50% of testosterone and 78% estradiol, leaving approximately 2% free Heyns and De Moor^(^ ^72^ ^)^. However, numerous conditions affect this binding including estrogens, thyroid hormone, adiponectin, and PPARγ ligands that increase SHBG expression, and monosaccharides and proinflammatory cytokines that reduce its expression (reviewed in Hammond^(^ ^74^ ^)^). Thus, obesity (via decreased adiponectin), diabetes mellitus (via increased glucose), and inflammation can all alter SHBG levels. Similarly, liver disease and nephrotic syndrome can reduce SHBG levels via decreased production and urine losses, respectively, without obvious abnormalities in sexual phenotype. Moreover, a family has been described in which the male proband totally lacked measurable SHBG, but had normal sexual development and free testosterone levels.^(^ ^75^ ^)^ Thus, measurement of total testosterone (and estradiol) can be misleading in these circumstances. However, though free testosterone is likely to be a better assessment of androgen status,^(^ ^66^, ^74^, ^76^ ^)^ free testosterone measurements are at this point not totally reliable. As for the other hormones in this review, equilibrium dialysis followed by liquid chromatography/mass spectrometry is the gold standard, but it is not suitable for high throughput.^(^ ^77^ ^)^ Direct analog tracer‐based immunoassays are not considered reliable and their use not recommended.^(^ ^78^ ^)^ Calculation of the free levels based on affinity constants for SHBG and albumin depend on the invariance of these binding constants,^(^ ^79^ ^)^ an assumption that is invalid given that the two sites for testosterone on the SHBG homodimer differ and show allosteric interaction.^(^ ^80^ ^)^ Thus, the ratio of testosterone to SHBG can alter the apparent affinity of these sites.^(^ ^81^ ^)^ Moreover, such interactions in binding sites may also apply to the binding of testosterone to albumin.^(^ ^82^ ^)^ Furthermore, there are numerous polymorphisms in SHBG that influence its affinity for sex steroids (reviewed in Hammond^(^ ^74^ ^)^). Finally, a direct comparison between calculated values and those determined by equilibrium dialysis demonstrated major discrepancies.^(^ ^83^ ^)^ Therefore, the routine use of directly measured free testosterone remains desirable yet problematic and cannot be replaced by calculated values.

SHBG circulates as a 90 kDa dimer. Calcium is required for the dimerization. It is 80% saturated with testosterone in men, 20% saturated in women. The SHBG gene is located on chromosome 17. Hepatic nuclear factor 4α (HNF4α) is its main transcription factor regulator.^(^ ^84^ ^)^ Regulation of HNF4a levels appears to be the means by which proinflammatory cytokines,^(^ ^85^, ^86^ ^)^ adiponectin,^(^ ^87^ ^)^ and thyroid hormone^(^ ^88^ ^)^ alter SHBG expression.

Although transport of the sex steroids is the major function of SHBG, SHBG can leave the vascular system with accumulation in extravascular tissues such as the endometrium and epididymis, but the biologic significance of this is not clear.^(^ ^89^ ^)^ As noted previously, megalin may transport SHBG into some cells,^(^ ^7^ ^)^ and megalin KO mice have abnormalities in the reproductive tract. Changes in SHBG levels have been associated with a variety of clinical conditions including osteoporosis^(^ ^90^ ^)^ and risk of diabetes mellitus,^(^ ^91^ ^)^ suggesting that SHBG may have functions beyond sex steroid transport, but these functions are not defined.

## Cortisol‐Binding Globulin and Free Cortisol

CBG is the major transport protein for cortisol, binding approximately 90% of the total cortisol in blood. Albumin carries about 6% to 7% with the free levels about 4% of the total, although this ranges to 10% in some reports. CBG also transports aldosterone, but with lower affinity such that CBG accounts for 21% of bound aldosterone with albumin transporting approximately 42%, leaving 37% free.^(^ ^92^ ^)^ CBG also transports a small (17%) fraction of progesterone, which is mostly carried by albumin.^(^ ^93^ ^)^ As noted in Table 1, the affinities of CBG and albumin for these hormones are substantially lower than that of DBP and TBG for their respective hormones. As for the other hormone‐binding globulins, the role of CBG beyond hormone transport is not clear. Families null for CBG do not show major clinical syndromes indicating cortisol deficiency, although hypotension and fatigue have been noted.^(^ ^94^, ^95^ ^)^ These clinical observations are consistent with the free hormone hypothesis, that CBG is not required for entry into cells. A list of mutations in CBGs affecting both levels and affinity for cortisol is found in Hammond^(^ ^74^ ^)^ and Gagliardi and colleagues,^(^ ^94^ ^)^ many of which have minimal clinical impact. However, mice with CBG null mutations appear to have increased sensitivity to an acute inflammatory challenge.^(^ ^96^ ^)^ CBG is cleaved by neutrophil elastase, conceptually providing increased free cortisol at infection sites,^(^ ^97^ ^)^ which may contribute to the altered response to inflammation in CBG null mice.

CBG is a 383 amino acid glycoprotein that circulates as a monomer of 50‐60 kDa according to its level of glycosylation. Although the liver is the main source of CBG, other tissues such as the pancreas and kidney express CBG at least in mice during development, but apparently these tissues do not contribute to the circulating levels, and the role of CBG is unclear in these tissues.^(^ ^98^ ^)^ CBG is a member of the serine protease inhibitor class located on chromosome 14 with homology to alpha 1 antitrypsin, but has no protease activity of its own.^(^ ^99^ ^)^ It has a single binding site for the steroids it transports. CBG levels are increased by estrogen^(^ ^100^ ^)^ and pregnancy,^(^ ^101^ ^)^ but are reduced by proinflammatory cytokines^(^ ^102^ ^)^ and critical illness,^(^ ^103^ ^)^ raising the concept that free cortisol measurements as in the saliva or urine might be a better way to assess cortisol status during acute illness.^(^ ^104^ ^)^ Measurement of free cortisol in blood has received little attention, nor have efforts to calculate free cortisol from published affinity constants been widely made.

## Summary and Conclusions

The vitamin D metabolites, thyroid hormones, sex steroids, and glucocorticoids/mineralcorticoids are lipophilic hormones whose principal actions involve nuclear hormone receptors. Thus they need to enter cells. But because of their lipophilic nature, they are transported in the liquid milieu of the blood by proteins both specific for the hormone such as DBP, TBG, SHBG, and CBG and the nonspecific protein albumin. The free hormone hypothesis postulates that only the unbound or free hormone crosses the cell membrane. To the extent this is valid, this raises the question whether it is the free concentration that should be measured rather than the total, especially in circumstances when the levels and/or affinities of the binding proteins are altered both physiologically (eg, pregnancy), pathophysiologically (liver disease, nephrotic syndrome, acute illness), or by genetic mutations. In some cases, the binding protein may play a role in transcellular transport as in the role of megalin in the kidney to reclaim DBP bound to 25OHD in the glomerular filtrate or in SHBG bound to sex steroids in reproductive tissues. But for the most part, the free hormone hypothesis remains validated as best demonstrated by the relative lack of clinical phenotypes in families or animals lacking the specific binding protein. That said, one cannot conclude that these binding globulins have no role other than hormone transport as is well‐demonstrated by the ability of DBP to serve as an actin scavenger or as part of the macrophage activating factor, TTR to transport thyroid hormones into the cerebrospinal fluid, possible role of SHBG to transport sex steroids into the placenta, or CBG to deliver increased cortisol to sites of infection (via cleavage by neutrophil elastase). However, a major problem with strongly advocating for measurement of free hormone levels is a lack of well‐accepted means of making these measurements short of equilibrium dialysis and LC–MS of the dialysate containing the free hormone. Although FT4 is now in widespread use, other free hormone measurements remain less used because of concerns regarding their validity. This is changing, and with harmonization of the existing and future assays, I anticipate that free hormone measurements will become routinely used to assess hormone status for the vitamin D metabolites, sex hormones, and for adrenal assessment much as we use FT4 to help assess thyroid status. This will be driven by the many conditions in which total hormone measurements are misleading because of changes in the affinity and level of their binding proteins.

## Disclosures

The author has nothing to disclose.

### Peer Review

The peer review history for this article is available at https://publons.com/publon/10.1002/jbm4.10418.
